# FBXO22 promotes the development of hepatocellular carcinoma by regulating the ubiquitination and degradation of p21

**DOI:** 10.1186/s13046-019-1058-6

**Published:** 2019-02-26

**Authors:** Long Zhang, Jin Chen, Deng Ning, Qiumeng Liu, Chao Wang, Zhaoqi Zhang, Liang Chu, Chengpeng Yu, Hui-fang Liang, Bixiang Zhang, Xiaoping Chen

**Affiliations:** 1 Hepatic Surgery Center, Tongji Hospital, Tongji Medical College, Huazhong University of Science and Technology, Clinical Medicine Research Center for Hepatic Surgery of Hubei Province, Key Laboratory of Organ Transplantation, Ministry of Education, NHC Key Laboratory of Organ Transplantation, Key Laboratory of Organ Transplantation, Chinese Academy of Medical Sciences, Wuhan, Hubei 430030 People’s Republic of China; 2 Department of Hepatobiliary Surgery, The First Affiliated Hospital, College of Medicine, Shihezi University, Shihezi, Xinjiang 832008 People’s Republic of China; 3 Department of Biliary and Pancreatic Surgery, Tongji Hospital, Tongji Medical College, Huazhong University of Science and Technology, Wuhan, Hubei 430030 People’s Republic of China

**Keywords:** FBXO22, Ubiquitination, p21, HCC

## Abstract

**Background:**

Deregulation of ubiquitin ligases is related to the malignant progression of human cancers. F-box only protein 22 (FBXO22), an F-box E3 ligase, is a member of the F-box protein family. However, the biological function of FBXO22 in HCC and the underlying molecular mechanisms are still unclear. In this study, we explored the role of FBXO22 in HCC and its mechanism of promoting tumor development.

**Methods:**

We examined the expression of FBXO22 in normal liver cell lines, HCC cell lines, HCC tissue microarrays and fresh specimens. The correlation between FBXO22 and clinical features was analyzed in a retrospective study of 110 pairs of HCC tissue microarrays. Univariate and multivariate survival analyses were used to explore the prognostic value of FBXO22 in HCC. At the same time, the correlation between the FBXO22 and p21 was also studied in HCC samples. Knock-down and overexpression experiments, CHX and Mg132 intervention experiments, ubiquitination experiments, rescue experiments and nude mouse xenograft models were used to determine the potential mechanism by which FBXO22 promotes tumorigenesis in vitro and in vivo.

**Results:**

The expression of FBXO22 in HCC tissues was significantly higher than in normal liver tissues. The overall survival rate and disease-free survival time of patients with high expression of FBXO22 were significantly shorter than those of patients with low expression of FBXO22. The high expression of FBXO22 in HCC tissues were significantly correlated with serum AFP (*p* = 0. 003, Pearson’s chi-squared test), tumor size (*p* = 0. 019, Pearson’s chi-squared test) and vascular invasion (*p* = 0. 031, Pearson’s chi-squared test). Especially, Multivariate analysis showed that tumor size and the expression of FBXO22 were independent prognostic indicator of OS (95% CI: 1.077–5.157, *P*<0.05). Correlation analysis also showed that FBXO22 was negatively correlated with p21 in tissue microarrays (r = − 0.3788, *P*<0.001, Pearson correlation) and fresh specimens (r = − 0.4037, *P*<0.01, Pearson correlation). Moreover, both in vitro and in vivo experiments showed that knocking down FBXO22 expression could inhibit cell proliferation, while overexpression of FBXO22 promoted tumor formation. Furthermore, we identified that FBXO22 interacts with p21 by regulating protein stability and by influencing the ubiquitination process. A knockdown of FBXO22 decreased the ubiquitylation of p21, while overexpression enhanced it.

**Conclusions:**

This study uncovered a new mechanism by which FBXO22 functions as an oncogene in HCC pathogenesis and progression by mediating the ubiquitination and degradation of p21. It was also found that tumor size and the expression of FBXO22 were independent prognostic indicator of OS and the expression of FBXO22 and p21 was negatively correlated in clinical samples. Our findings present a new perspective for understanding the development of HCC, which may provide a new target for the treatment and management of this challenging cancer.

**Electronic supplementary material:**

The online version of this article (10.1186/s13046-019-1058-6) contains supplementary material, which is available to authorized users.

## Introduction

Hepatocellular carcinoma (HCC) is one of the most common malignancies worldwide, and is a highly lethal cancer since it is frequently diagnosed at an advanced stage [1]. Relevant evidence suggests that the pathogenesis of HCC involves epigenetic and genetic changes [2]. However, the molecular mechanism of HCC development is still incompletely understood.

Ubiquitin is a small regulatory protein that is ubiquitous in eukaryotic cells [3]. The ubiquitination via the E1-E2-E3 (ubiquitin-activation enzyme, ubiquitin-conjugating enzymes, ubiquitin-protein ligases) three-step cascade reaction is the most common, diverse and multifunctional post-translational protein modification in cells, and is involved in various life activities such as protein hydrolysis and signal transduction [4]. E3 ubiquitin ligase is recognized as the most important component of the ubiquitination process due to its specific recognition of substrates. Depending on the structure and function, E3 ubiquitin ligases can be divided into four families – the plant homeodomain (PHD) finger family, the homologous to E6-associated protein C-terminus (HECT) family, the ring-finger family and the U-box family. The SKP1-Cullin1-F-box protein (SCF) complex is the most important and most studied E3 ubiquitin ligase in the ring-finger family. It consists of four components: the adaptor protein SKP1, the scaffold protein Cullin1, the RING-finger protein RBX1 and an F-box protein. The first three proteins constitute a stable single frame for binding to different F-box proteins, while the F-box protein specifically recognizes different substrate proteins via the C-terminus [5, 6]. According to the differences in the secondary structure of their C-terminus, F-box proteins can be divided into three categories – FBXL, FBXW and FBXO [7].

There are few reports on the roles played by F-box only protein 22 (FBXO22), a member of the FBXO protein family, in biological activities. FBXO22 mediates the degradation of kruppel-like factor 4 (KLF4) [8] and the lysine-specific demethylase 4A (KDM4A) [9], as well as the methylation of p53 [10]. In addition, a recent study has shown that FBXO22 plays a dual role in controlling breast cancer growth and metastasis. FBXO22 proliferative role in primary breast cancer, and degrades SNAIL through ubiquitination to exert an anti-metastatic effect [11]. However, the biological function of FBXO22 in HCC and the underlying mechanisms remain unclear.

In this study, we investigated the effects of FBXO22 on HCC progression and the underlying mechanism. We demonstrated that FBXO22 interacts with p21 and regulates the protein level of p21 through the ubiquitination pathway in HCC. Our data suggest that FBXO22 functions as an oncogene in HCC pathogenesis and progression by mediating the degradation of p21.

## Materials and methods

### Patients and HCC tissue specimens

A total of 50 paired specimens of tumor and adjacent non-tumor tissues were collected from 50 HCC patients (45 men and 5 women; median age, 47 years; age range, 26–79 years) who underwent hepatectomy at the Hepatic Surgery Center, Tongji Hospital of Huazhong University of Science and Technology (HUST) (Wuhan, China). Matched fresh specimens of HCC tissues and adjacent non-tumorous liver tissue were lysed separately for western blot analysis. A tissue microarray of 110 pairs of primary HCC tissues with their clinical and prognosis data were acquired from the specimen library of the Hepatic Surgery Center, Tongji Hospital of Huazhong University of Science and Technology. The samples were obtained in surgeries from 2012/2/16 to 2014/4/1, and the database has been updated every month since 2012/2/16.

### Cell lines and culture conditions

All cell lines (HL-7702, HepG2, Huh7, Hep3B, Bel-7402, HLF, LM3 and 293 T) were purchased from the China Center for Type Culture Collection (Wuhan, China) and cultured in Dulbecco’s modified Eagle’s medium (Invitrogen Corporation, Carlsbad, CA, USA) supplemented with 10% fetal bovine serum (Life Technologies Inc., Gibco/Brl Division, Grand Island, NY, USA) in a humidified atmosphere comprising 5% CO_2_ at 37 °C. HepG2, Hep3B and 293 T lines were authenticated by comparative genomic array hybridization or short tandem repeat DNA profiling according to the ATCC database, which were performed within less than 10 passages after authentication and less than 20 passages after receipt from commercial suppliers. Other cell lines were identified via STR genotyping test method by Wuhan Genecreate Biological engineering Co., Ltd.

### Chemicals, inhibitors and antibodies

Puromycin and G418 were purchased from Cayman Chemical Company (Ann Arbor, MI, USA). NEM and doxorubicin was purchased from Sigma-Aldrich Corporation (St. Louis, MO, USA). CHX and Mg132 were obtained from MedChemExpress (Shanghai, China). The following antibodies were used for either western blotting or immunohistochemical analysis: FBXO22 (13606–1-AP, Protein-tech, Wuhan, China), CDK2 (SC-748, Santa Cruz Biotechnology, CA, USA), CDK4 (SC-260, Santa Cruz Biotechnology, CA, USA), cyclinE1 (EP435E, Abcam, Cambridge, MA, USA), and GAPDH (KC-5G4; Kang Chen Bio-tech, Inc., Shanghai, China). The following antibodies were purchased from Cell Signaling Technology, Inc. (Beverly, MA, USA): Akt (C67E7), phospho-Akt (Ser473) (D9E), Erk (137F5), p38 (D13E1), phospho-p38 (3D7), p70s6k (49D7), phospho-p70s6k (1A5), cyclin B1 (V152), cyclin D1 (92G2) and p21(12D1).

#### Western blot analysis

Western blot analysis was performed as described previously [12], and the results were quantified using the image analysis tool ImageJ (National Institutes of Health, Bethesda, MD, USA). Each experiment was repeated three times.

### Real-time PCR

Real-time PCR was performed as described previously [13]. The following primers were used for FBXO22: 5′-CGGAGCACCTTCGTGTTGA-3′ (forward) and 5′-CACACACTCCCTCCATAAGCG-3′ (reverse), p21:5′-TGTCCGTCAGAACCCA TGC-3′ (forward) and 5′-AAAGTCGAAGTTCCATCG CTCAG-3′ (reverse), p53: 5′-CAGCACATGACGGAGGTTGT-3′ (forward) and 5′-TCATCCAAATACTCCAC ACGC-3′ (reverse) and GADPH: (forward) 5’-GACAAGCTTCCCGTTCTCAG-3′ and (reverse) 5’-GAGTCAACGGATTTGGT CGT-3′. The relative expression of each gene was analyzed using the 2^-ΔΔCt^ method [14]. The experiments were done in triplicate. The primers were all synthesized by TSINGKE Company (Beijing, China).

### Plasmids, lentiviral vectors and stable cell lines

Full-length human FBXO22 cDNA was amplified by PCR and subcloned into the lentiviral vector pBABE-puro (plasmid # 1764; Addgene, Cambridge, MA, USA) to establish LM3 and Hep3B cell lines that stably overexpress FBXO22. The target sequences corresponding to FBXO22 were used to establish stable HLF and HepG2 FBXO22 knockdown cell lines. In addition, the target sequences corresponding to p21 (shRNA: GATGGAACTTCGACTTTGT) were subcloned into the lentiviral vector GV152 (vector containing a neomycin resistance gene; Genechem Co. Ltd., Shanghai, China) to establish stable HLF and HepG2 cell lines with FBXO22 knocked down.

Briefly, DNA fragments (FBXO22 shRNA#1 CAAGTAGTCAGCACTTTCA, FBXO22 shRNA#2 GGAATTGTAGTGACTCCAATG) were subcloned into the lentiviral vector pLKO.1 puro (plasmid # 10787; Addgene). The plasmids pMD2.G and psPAX2 were gifts from Didier Trono (plasmids # 12259 and #12260; Addgene). For plasmid transfection, 293 T cells were plated in 6-cm dishes and co-transfected with target plasmids (1 μg) and virus packaging plasmids (pMD2.G 0.25 μg, psPAX2 0.75 μg) using Lipofectamine2000 (Invitrogen, USA) or Xtreme HP (Roche, Basel, Switzerland). Eight hours after transfection, cells were transferred to fresh medium-containing 10% FBS and incubated for 48-72 h. The lentivirus-containing supernatants were collected, passed through a 0.45 μm filter (PALL, Port Washington, NY, USA), and used for infection of the cells. The HCC cells were transfected with the lentivirus in the presence of polybrene (8 μg/ml; Sigma, St Louis, MO, USA). At 48 h after infection, the cells were selected using growth medium containing 5 μg/ml puromycin for 7 days or 400 μg/ml G418 for 14 days. GV152 transfection of the indicated HCC cell lines followed the manufacturer’s instructions. The efficiency of overexpression and knockdown was verified by RT-PCR or western blot analysis.

### Cell viability assay

For the Cell Counting Kit-8 (CCK8) assay (Sigma-Aldrich Corporation, St. Louis, MO, USA), cells were seeded into 96-well plates for 5 to 6 days with replacement of the culture medium every 2 days. Cell Counting Kit-8 solution was added to the wells of the plates and for 1–2 h to test the optical density (OD) value at 450 nm (Elx 800; BioTek Instruments, Inc., Winooski, VT, USA). For the colony formation assay, 500 cells/well for HLF and HepG2, or 2500 cells/well for Hep3B were seeded into six-well plates with replacement of the culture medium every 2 days. After 2 weeks, the plates were stained with 1% crystal violet (Sigma-Aldrich Corporation) and photographed. Colonies were counted and analyzed using the Alpha Innotech Imaging system (Alphatron Asia Pte, Ltd., Singapore). Each experiment was repeated three times.

### Immunohistochemistry (IHC)

IHC analysis was performed as described previously [15]. The microscopic examination of each point of the tissue microarray was performed at the same incident light intensity and compensation intensity. The total score for each point was the product of the staining intensity score and the stained positive cells score. The rules of the staining intensity scoring were as follows: 0 points (Negative); 1 point (Light brown); 2 points (Brown); 3 points (Dark brown). The rules of stained positive cells scoring were as follows: 0 points (0%); 1 point (10–25%); 2 points (26–50%); 3 points (51–75%); 4 points (76–100%). We defined the assay as positive if the total score was greater than or equal to 6 points, otherwise we defined it as negative. The total scoring of the tissue chip was independently completed by two pathologists who had no knowledge of the patient’s clinical case data. The FBXO22 antibody was diluted 1:150. Each experiment was repeated three times.

### Tumorigenicity assay

Four-week-old male BALB/c (nu/nu) mice were raised under specific pathogen-free conditions. All animals were cared for according to the Guide for the Care and Use of Laboratory Animals [16]. The experimental protocol was approved by the Committee on the Ethics of Animal Experiments of the Tongji Medical College, HUST. For the xenograft tumorigenicity assay, 1 × 10^6^ tumor cells suspended in phosphate-buffered saline were injected subcutaneously into the flanks of the nude mice (*n* = 6 per group). For intrahepatic orthotopic tumor experiment, 1 × 10^6^ cells were injected into the liver of the tested nude mouse (*n* = 5 per group) and postoperative care was performed. After surgery, mice were placed in a cage under a heater until recovered from anesthesia. Analgesia was administrated during the first 2–3 postoperative days. All of the mice were sacrificed 4 weeks after injection. The length and width of the tumors were measured with a Vernier caliper after sacrifice, and the tumor volume was calculated according to the formula V = L × W^2^ × 0.5. After the experiment, the livers excised from the mice were fixed in 4% paraformaldehyde.

### Immunoprecipitation and immunoblotting analyses

Cells were treated with 5 μM MG132 for 6 h before harvesting and subsequently lysed in IP lysis buffer (50 mM Tris-HCl, pH 7.4, 150 mM NaCl, 1 mM EDTA, 1% NP-40 and 10% Glycerin) containing protease inhibitors and phosphatase inhibitor (Roche). For the western blot analysis, coprecipitates or total cell lysates were separated by SDS-PAGE and transferred to PVDF membranes (ISEQ00010, Millipore, Merck KGaA, Germany). The membranes were blocked with 5% skim milk and reacted with primary antibodies at 4 °C overnight. Secondary antibodies including horseradish peroxidase-conjugated goat anti-mouse IgG and goat anti-rabbit IgG (Santa Cruz Biotechnology, CA, USA, as a negative control) were reacted at room temperature for 1 h, after which the reactive bands were visualized using the image analysis tool ImageJ (National Institutes of Health, Bethesda, MD, USA).

### Ubiquitination assay

The cells were treated with 20 μM MG132 for 4 h before harvesting, after which they were washed with PBS and lysed with IP lysis/wash buffer with protease inhibitor and phosphatase inhibitor (Roche) and 10 μM N-ethylmaleimide (NEM; Sigma, St Louis, MO, USA) on ice for 30 min. The cleared lysates were quantified, and an equal amount of each lysate was used for immunoprecipitation with protein A/G agarose (Sigma) pre-bound with the specified antibodies. The resin beads were washed with lysis buffer, and samples were eluted. The eluted fraction was further separated on an SDS-PAGE gel. Subsequent immunoblotting was performed using the indicated antibodies using the enhanced chemiluminescence-detection system (BIO-RAD, CA, USA).

### Protein half-life assay

Cells were treated with cycloheximide (10 μM) for various periods of time to block protein synthesis. Crude extracts were prepared, and the protein levels were assessed using western blot analysis.

### Flow cytometry

The antibody used in flow cytometry was Annexin V-FITC (Abcam Inc., Cambridge, MA). After digestion, cells were adjusted to a density of 1 × 10^5^ cells/mL, and incubated with fluorescent antibody and propidium iodide (PI) staining in the dark at room temperature for 30 min. After incubation, cells were centrifuged at 200 *g* and then resuspended and analyzed with a flow cytometer (BD Bioscience, San Jose, CA).

### Statistical analysis

Data were recorded as the means ± standard deviation (SD). Survival analysis was analyzed using Kaplan-Meier method. Association between FBXO22 and p21 expression in HCC tissues was calculated using Pearson correlation test. The χ^2^ test was performed to analyze the relationship between FBXO22 expression and the clinicopathological characteristics. Based on the variables selected on univariate analysis, the multivariate Cox proportional hazards model was used to determine the independent prognostic factors of HCC. The differences between the groups were undertaken using the Student two-tailed t test and one-way ANOVA. A *p* < 0.05 was considered statistically significant. The statistical analysis and figure generation were performed using Prism 6.0 software (GraphPad Software, Inc., La Jolla, CA, USA) and SPSS version 25.0 (IBM).

## Results

### Association of FBXO22 expression with clinical outcomes of HCC patients

**Fig. 1:**
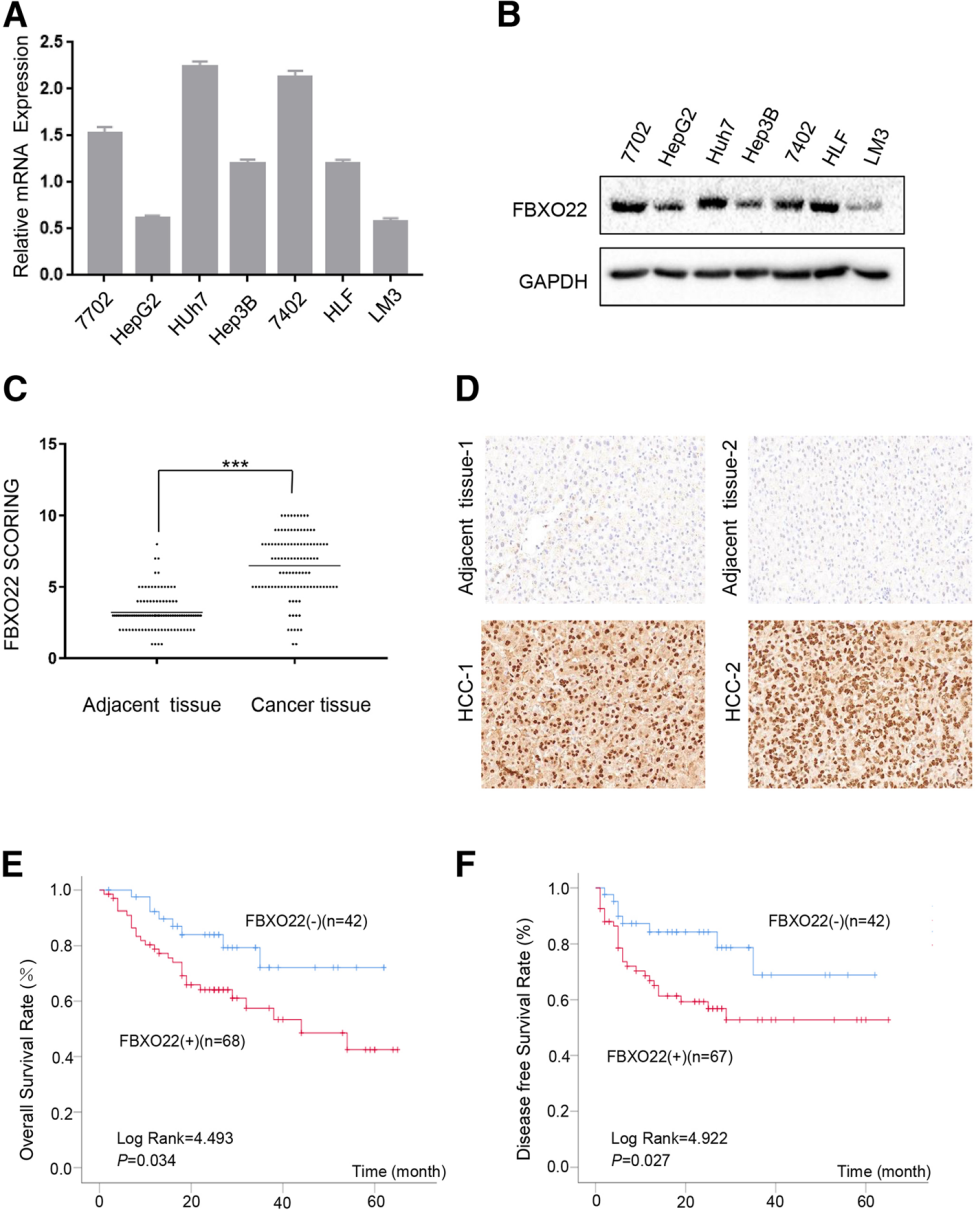
FBXO22 is overexpressed in HCC. (**a**) Real time PCR analysis of FBXO22 expression in one normal hepatic cell line and six HCC cell lines. GAPDH was used as a loading control. (**b**) Western blot analysis of FBXO22 expression in one normal hepatic cell line and six HCC cell lines. GAPDH was used as a loading control. (**c**) Dot chart of the expression of FBXO22 in 110 pairs of HCCs and matched tissues detected by IHC. The results are expressed as means ±SD. (**d**) Representative images of IHC staining with anti-FBXO22 (400×, magnification). (**e**) Kaplan-Meier overall survival curve of two HCC groups: FBXO22 (+), patients with high FBXO22 expression; FBXO22 (−), patients with low FBXO22 expression. (**f**) Kaplan-Meier disease-free survival curves of the two HCC groups: FBXO22 (+), patients with high FBXO22 expression; FBXO22 (−), patients with low FBXO22 expression

The expression levels of FBXO22 in one immortalized liver cell line and six HCC cell lines were determined by reverse transcription-PCR (RT-PCR) and western blot analysis (Fig. 1a and b, respectively). Because lower expression levels of FBXO22 were observed in LM3 and Hep3B cells, these two cell lines were used for the FBXO22 overexpression experiments. HLF and HepG2 cells were used for FBXO22 silencing because their FBXO22 expression levels are relatively high. To determine the clinical significance of FBXO22 expression in patients with HCC, we analyzed FBXO22 expression levels by IHC on a tissue microarray containing 110 pairs of HCC samples with available clinical follow-up information. The expression levels of FBXO22 among 110 HCC tumor samples and adjacent non-tumor tissues was compared using the IHC results. The results indicate that FBXO22 is overexpressed in tumors compared to adjacent non-tumor tissues (*p* < 0.001, paired Student’s *t*-test; Fig. 1c). Semiquantitative analysis revealed high expression of FBXO22 in 61.8% (68/110) of HCC patients, while 38.2% (42/110) of patients had low FBXO22 expression (Fig. 1d). Moreover, the expression levels of FBXO22 were associated with serum AFP (*p* = 0. 003, Pearson’s chi-squared test), tumor size (*p* = 0. 019, Pearson’s chi-squared test) and vascular invasion (*p* = 0. 031, Pearson’s chi-squared test). However, there was no statistical difference between FBXO22 expression levels and sex, age, ALT, AST, tumor number, BCLC stage, TNM stage, differentiation, tumor capsule and recurrence (Table 1). This may be due to the limited sample size and the fact that only early-stage patients who were eligible for surgical treatment were included in this study. On univariate survival analysis, the factors significantly associated with OS were serum AFP, tumor size, BCLC stage, TNM stage, differentiation, vascular invasion and positive expression of FBXO22. The patients with FBXO22 positivity showed a 2.275-folds (95% CI: 1.036–4.996, *P*<0.05) greater risk of death. Multivariate analysis was carried out on the factors significantly related to OS by univariate analysis. Multivariate analysis showed that tumor size and the positive expression of FBXO22 were independent prognostic indicator of OS. The FBXO22 positively indicated a 2.357-folds (95% CI: 1.077–5.157, *P*<0.05) greater risk of death (Table 2). Survival analysis using the Kaplan-Meier method revealed that patients with low FBXO22 expression had better overall survival (OS) (Long Rank = 4.493, *p* = 0.034, Fig. 1e) and disease-free survival (DFS) (Long Rank = 4.922, *p* = 0.027, Fig. 1f). These results indicate that low expression levels of FBXO22 are associated with better prognosis of patients with HCC.

**Table 1:** Association of FBXO22 Upregulation with Clinicopathologic Features in 110 Primary HCCs

Features	Total	FBXO22 Expression		*P* value
		Normal Expression	Over Expression	
*Sex*				
Male	90	35	55	0.746
Female	20	7	13	
*Age (years)*				
≤ 50	55	22	33	0.695
>50	55	20	35	
*ALT*				
<70	100	37	63	0.420
≥ 70	10	5	5	
*AST*				
<70	96	37	59	0.839
≥ 70	14	5	9	
*Serum AFP (ng/ml)*				
<400	56	29	27	*0.003*
≥ 400	54	13	41	
*Tumor size (cm)* ^*^				
≤ 5	40	21	19	*0.019*
>5	70	21	49	
*Tumor number*				
Single	84	31	53	0.620
Multiple	26	11	15	
*BCLC stage*				
0 + A	70	31	39	0.140
B + C	40	12	28	
*TNM stage*				
I + II	81	34	47	0.171
III + IV	29	8	21	
*Differentiation*				
Well/moderate	71	27	44	0.964
Poor	39	15	24	
*Vascular invasion*				
Yes	88	38	50	*0.031*
No	22	4	18	
*Tumor capsule*				
Absent	53	16	37	0.096
Present	57	26	31	
*Recurrence*				
Yes	53	24	29	0.139
No	57	18	39	

NOTE. Statistical significance (P < 0.05) is shown in bold

^*^ Tumor size was measured by the length of the largest tumor nodule

**Table 2:** Clinicopathologic factors and their effect on overall survival by univariate and multivariate Cox proportional hazards regression analysis

Clinicopathological Features	Univariate Analysis			Multivariate Analysis		
	HR^a^	95%CI^b^	*P* value	HR^a^	95%CI^b^	*P* value
Sex	0.809	0.313–2.088	0.661			
Age (years)	1.159	0.602–2.232	0.659			
ALT	1.770	0.684–4.577	0.239			
Tumor number	1.153	0.542–2.455	0.712			
Tumor capsule	0.617	0.319–1.194	0.151			
*Serum AFP (ng/ml)*	2.806	1.379–5.706	*0.004*	1.502	0.642–3.516	0.348
*Tumor size (cm)* ^*^	3.333	1.387–8.014	*0.007*	2.955	1.376–6.346	*0.005*
*BCLC stage*	2.561	1.326–4.947	*0.005*	0.439	0.156–1.231	0.118
*TNM stage*	2.613	1.351–5.055	*0.004*	0.400	0.139–1.149	0.089
*Differentiation*	0.507	0.262–0.981	*0.044*	0.766	0.339–1.731	0.521
*Vascular invasion*	4.154	2.049–8.421	*0.000*	0.885	0.351–2.234	0.796
*FBXO22 overexpression*	2.275	1.036–4.996	*0.041*	2.357	1.077–5.157	*0.032*

a, hazard ratio; b, confidence interval

### FBXO22 promotes HCC cell proliferation in vitro and tumor growth in vivo

**Fig. 2:**
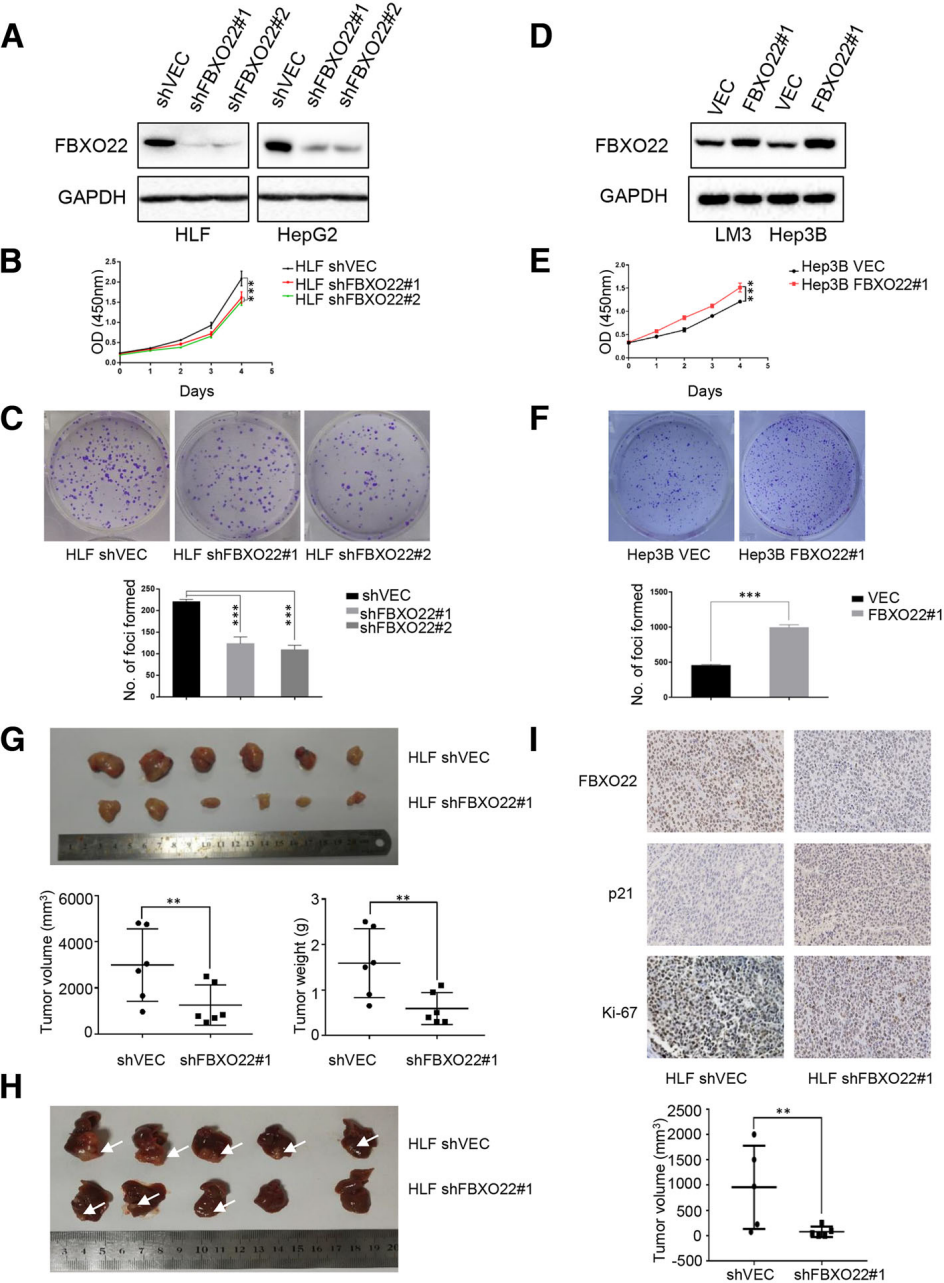
FBXO22 promotes proliferation and tumorigenesis of HCC cells in vitro and in vivo. (**a**) shFBXO22 effectively decreased FBXO22 expression in HLF and HepG2 cells. Transfection with scrambled shRNA (shvec) was used as negative control, and GAPDH was used as a loading control. Knockdown of FBXO22 expression effectively inhibited cell growth (**b**), foci formation (**c**), and tumor formation in nude mice (**g**, **h**), while FBXO22 was stably overexpressed in Hep3B and LM3 cells as detected by western blot analysis. Transfection with empty vectors (vector) were used as negative control, and GAPDH was used as a loading control (**d**). Overexpression of FBXO22 promoted cell growth (**e**) and foci formation (**f**). The results are expressed as the means ± standard error of the mean (SEM) of three independent experiments. Xenograft Tumor weight and tumor volume are shown as means ± SEM (**g**). The results are expressed as the means ± standard error of the mean (SEM) of three independent experiments. Orthotopic tumor volume are shown as means ± SEM (**h**). Representative IHC images of FBXO22, p21 and ki-67 expression in xenograft tumors (400×, magnification) (I)

To investigate the impact of FBXO22 on the malignant phenotypes of HCC cells, two short hairpin RNA (shFBXO22#1 and shFBXO22#2) specifically targeting FBXO22 was used to transfect HLF and HepG2 cells. Western blot analysis showed that the shRNA significantly down-regulated the expression of FBXO22 (Fig. 2a). Cell proliferation assays using the CCK-8 kit revealed that knockdown of FBXO22 slowed down the proliferation of HLF (*p*<0.01, Student’s *t*-test) and HepG2 cells (*p*<0.01, Student’s *t*-test) (Fig. 2b and Additional file 1: Figure S1A, respectively). Similarly, the colony formation assay demonstrated that knockdown of FBXO22 decreased colony formation of HLF (*p*<0.05, Student’s *t*-test) and HepG2 (*p*<0.01, Student’s *t*-test) cells (Fig. 2c and Additional file 1: Figure S1B, respectively).

To further confirm the effect of FBXO22 on the cells’ proliferation ability, a lentiviral FBXO22 expression vector was stably transduced into the two cell lines Hep3B and LM3. The empty lentivector was used as a control. Expression of FBXO22 was detected by western blot analysis (Fig. 2d). Overexpression of FBXO22 in Hep3B cells increased cell viability compared with control cells (Fig. 2e). The colony formation assay also showed that the foci formation frequency was obviously higher in FBXO22-overexpressing cells (*p*<0.01, Student’s *t*-test), with control cells (Fig. 2f). To investigate whether FBXO22 increased the tumorigenic capacity of HCC cells in vivo, HLF cells with stably knocked down FBXO22 expression were subcutaneously injected into the right dorsal flanks of 6-week-old BALB/c nude mice. Consistent with the in vitro results, xenograft tumors with knocked down FBXO22 expression grew slower than those carrying the empty vector (Additional file 1: Figure S1C). Moreover, the results indicated that tumors developed from HLF-shFBXO22 cells were significantly smaller (*p*<0.05, Student’s *t*-test) and lighter (*p*<0.05, Student’s *t*-test) than tumors derived from control cells (Fig. 2g), Similarly, orthotopic tumor developed from HLF-shFBXO22 cells were also significantly smaller (*p*<0.05, Student’s *t*-test) than tumors derived from control cells (Fig. 2h), which was related to the decreased cell proliferation rate due to the downregulation of FBXO22. Consistently, IHC staining showed low expression of FBXO22 and Ki-67 in the xenografts comprising shFBXO22-treated cells. However, the expression of p21 was increased compared to the control cells (Fig. 2i). These results were confirmed by repeated experiments using newly established stable HLF FBXO22 knockdown lines. Taken together, these results suggest that FBXO22 promotes HCC cell proliferation in vitro and tumor growth in vivo.

### FBXO22 regulates the protein levels of p21

**Fig. 3:**
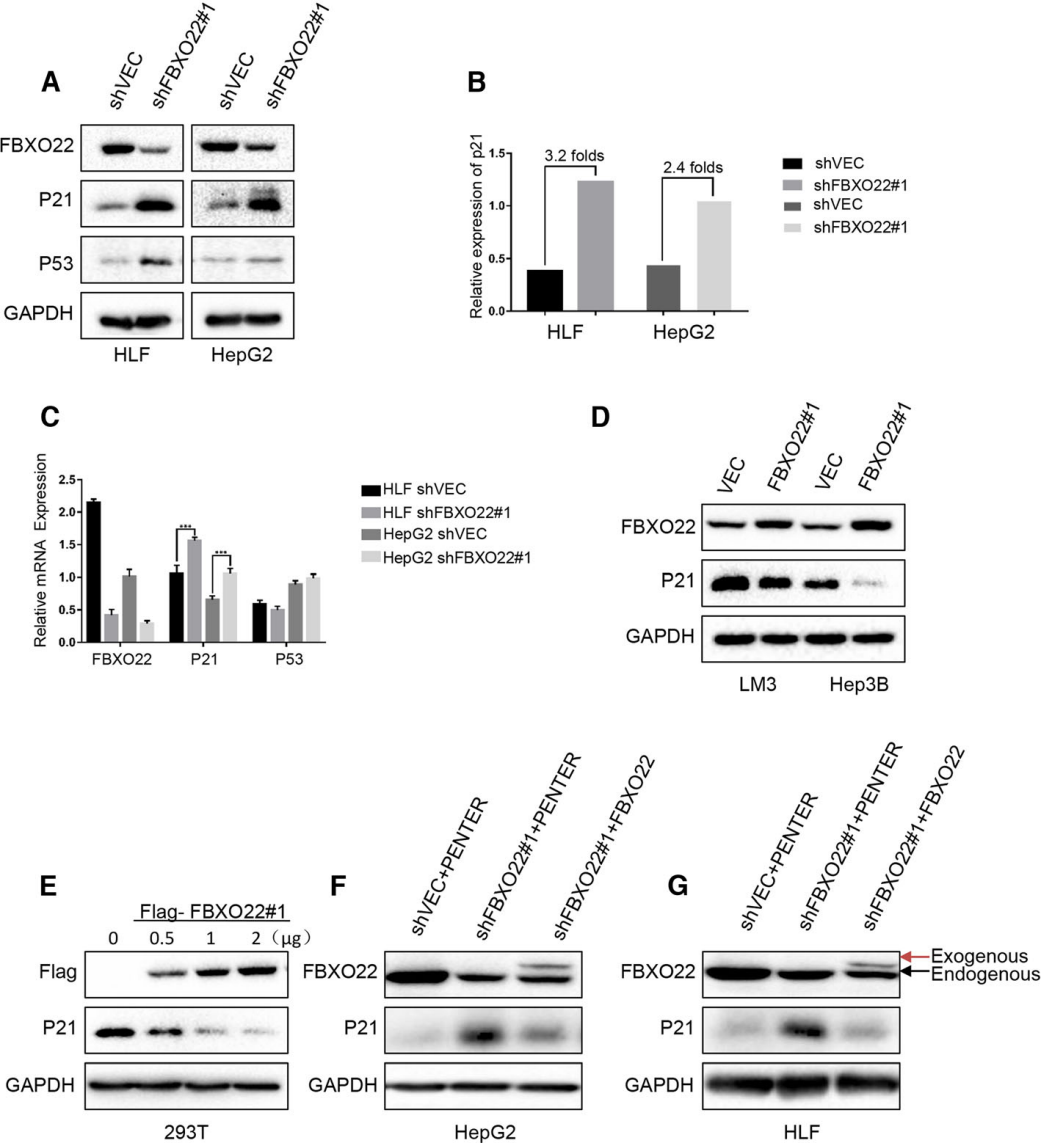
FBXO22 regulates the protein levels of p21. (**a**) HLF and HepG2 cells were transfected with the indicated constructs, total protein was extracted and subjected to western blotting using anti-FBXO22, anti-p21, and anti-GAPDH antibodies, respectively. (**b**) Quantification of the p21 levels relative to GAPDH expression (**c**) HLF and HepG2 cells were transfected with the indicated constructs, total RNA was extracted and subjected to real-time qPCR using primers specific for FBXO22, p21, or GAPDH. (**d**) Hep3B and LM3 cells were transfected with the indicated constructs, total protein was extracted and subjected to western blotting using anti-FBXO22, anti-p21, and anti-GAPDH antibodies, respectively. (**e**) 293 T cells were transfected with Flag-FBXO22 at different dosages. Total protein was extracted and subjected to western blotting using anti-Flag, anti-p21, and anti-GAPDH antibodies, respectively. (**f**, **g**) FBXO22-depleted cell lines were transfected with the indicated constructs, total protein was extracted and subjected to western blotting using anti-FBXO22, anti-p21, and anti-GAPDH antibodies, respectively

Protein-protein interactions are known to play key roles in regulating p21 levels. Therefore, we next investigated whether FBXO22 regulates the p21 protein level. Cells with stably knocked down FBXO22 expression were used to detect the gene’s effect on signaling pathways and cell cycle related proteins (Additional file 1: Fig. S1D). A recent study has shown that FBXO22 interacts with p53, which regulates the expression of p21 [10]. Consistent with these results, we found that depletion of FBXO22 also increased the endogenous p21 and p53 protein levels in our context (Fig. 3a). Western blot analysis revealed that the relative expression of p21 increased 3.2-fold in HLF and 2.4-fold in HepG2 cells (Fig. 3b). However, analysis by RT-PCR revealed that the upregulation of average p21 mRNA levels was very slightly (increased 0.4-fold in HLF (*p*<0.001, one-way ANOVA) and 0.6-fold in HepG2 (*p*<0.001, one-way ANOVA)) and the average p53 mRNA levels did not differ significantly (Fig. 3c). A slight change in p21 mRNA levels is unlikely to result in a significant change of p21 at the protein level, suggesting that p21 expression is regulated by post-transcriptional modification in HCC. Then we constructed the FBXO22 overexpressing cell line, FBXO22 was introduced into LM3 as well as Hep3B cells. Interestingly, the stable expression of exogenous FBXO22 led to a significant decrease of endogenous p21 protein (Fig. 3d). Correspondingly, an increase of FBXO22 expression caused a decline of p21 levels in a dose-dependent manner in 293 T cells (Fig. 3e). To investigate whether FIST-N and FIST-C domains were involved in the regulation of p21 expression. We constructed the Flag-FBXO22 plasmids with mutated FIST-N and FIST-C, and transferred them into 293 T cells according to different doses, respectively. It is interesting to note that the protein expression of p21 did not change significantly with the increase of transfection dose, which means that FBXO22 could not regulate p21 without FIST-N and FIST-C domains. It also shows that FIST-N and FIST-C domains are necessary for FBXO22 to regulate p21(Additional file 1: Figure S1E). In order to verify the presence or absence of off-target effects, we transfected the FBXO22-depleted cells with the FBXO22 plasmid (Figs. 3e and f), and found that the upregulation of p21 was abolished after the overexpression of FBXO22 in FBXO22-depleted cells.

### FBXO22 interacts with p21

**Fig. 4:**
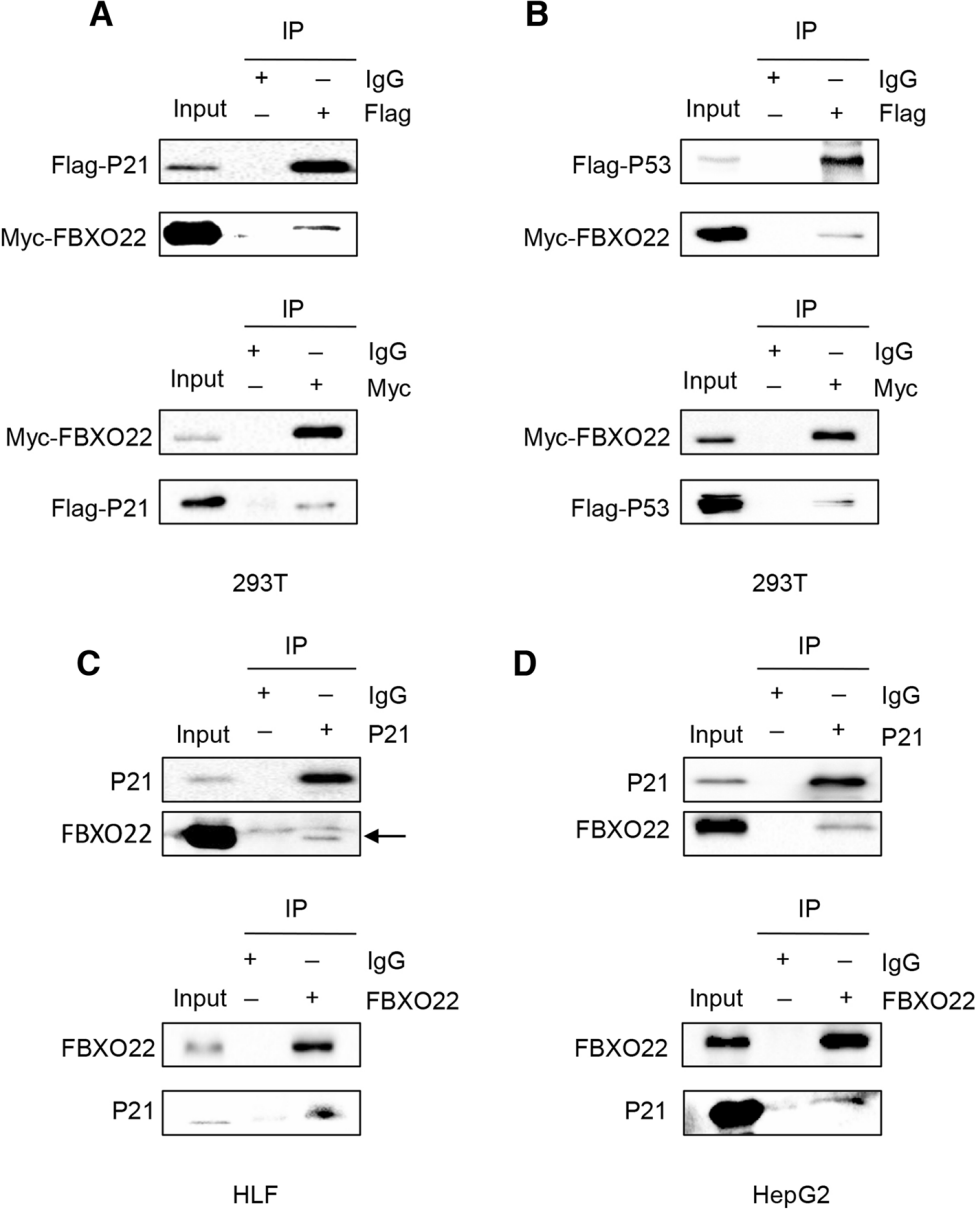
FBXO22 interacts with p21. (**a**) HEK293T cells transfected with vectors expressing Myc-FBXO22 and Flag-p21 were treated with Mg132 (5 μg/ml) for 6 h. The cell lysates were subjected to immunoprecipitation with anti-Flag or anti-Myc antibodies. (**b**) HEK293T cells transfected with vectors expressing Myc-FXBO22 and Flag-p53 were treated with Mg132 (5 μg/ml) for 6 h. The cell lysates were subjected to immunoprecipitation with anti-Flag or anti-Myc antibodies. (**c**) HLF cells were treated with Mg132 (5 μg/ml) for 6 h. The cell lysates were subjected to immunoprecipitation with anti-FBXO22 or anti-p21 antibodies. (**d**) HepG2 cells were treated with Mg132 (5 μg/ml) for 6 h. The cell lysates were subjected to immunoprecipitation with anti-FBXO22 or anti-p21 antibodies

To investigate the mechanism by which FBXO22 regulates the protein levels of p21, we next examined whether FBXO22 interacts with p21. Since FBXO22 has a weak influence on p21 at the transcriptional level, we speculated that this regulation may be mainly related to the posttranslational modification of p21. In addition, p53 and p21 are known to mutually interact [17]. The resulting p53/p21 complex, which can regulate cancer cell invasion or death, is a functional unit that acts on multiple cell components [18]. Therefore, we speculated that FBXO22 may interact with p21 to regulate its protein level. In order to confirm this assumption, HEK293T cells were transfected with Myc-FBXO22 in combination with Flag-p21 or Flag-p53, and then subjected to reciprocal IP assays with an anti-Flag or anti-Myc antibody. Immunoblotting showed that Myc-FBXO22 interacts with Flag-p21 and Flag-p53 (Fig. 4a and b, respectively). Importantly, an interaction between FBXO22 and p21 at the endogenous protein level was validated in HLF and HepG2 cells by co-immunoprecipitation with an anti-FBXO22 and an anti-p21 antibody (Fig. 4c and d, respectively).

### FBXO22 ubiquitylates p21 via its F-box domain

**Fig. 5:**
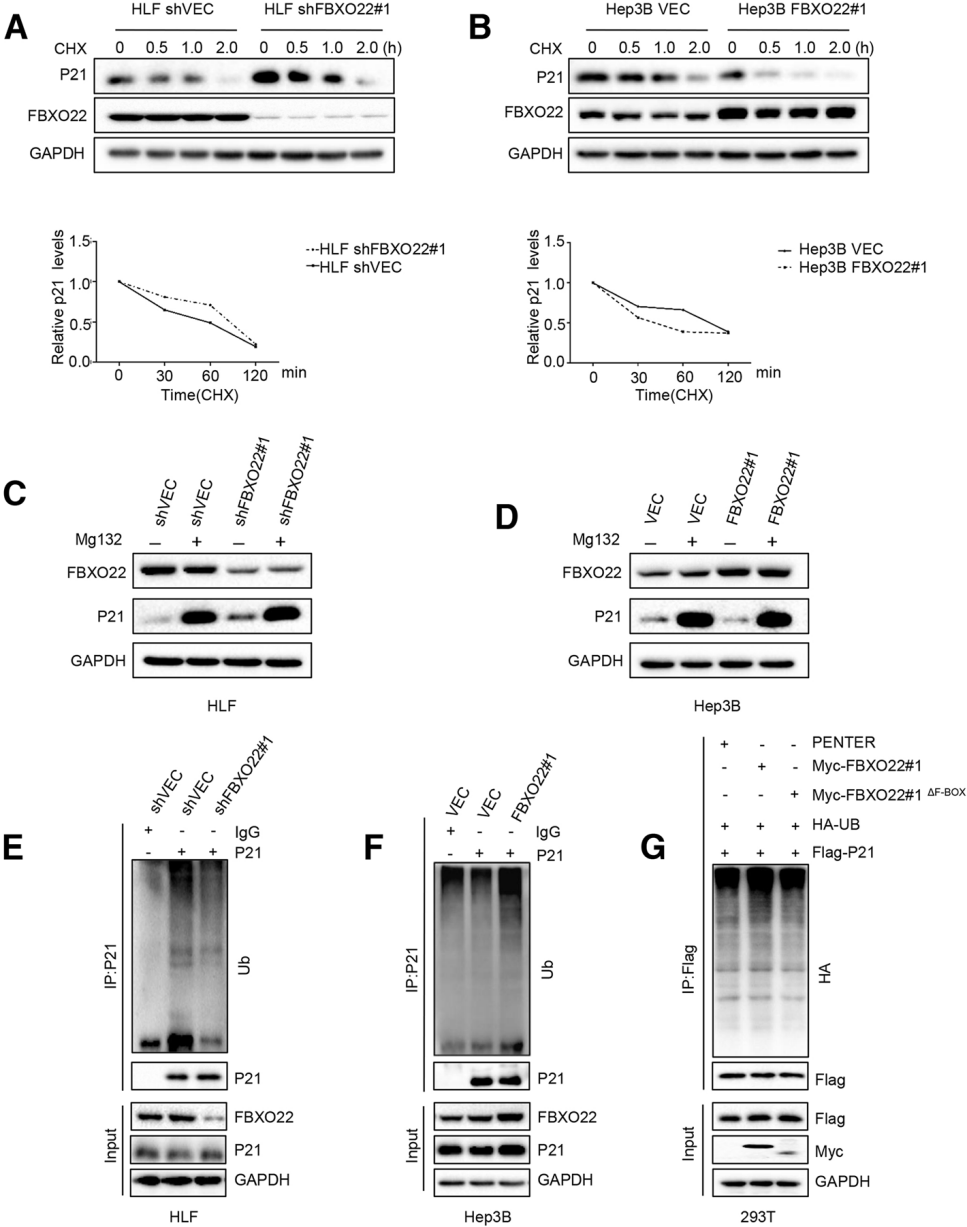
FBX022 ubiquitinates p21 via the F-box domain (**a** and **b**) HLF and Hep3B were treated with CHX (10 μM), collected at the indicated time points, and immunoblotted for FBXO22, p21 and GAPDH. Quantification of the p21 levels relative to GAPDH expression is shown. (**c** and **d**) HLF and Hep3B cells were treated with Mg132 (10 μg/ml) for 4 h, total protein was extracted and subjected to western blotting using anti-Flag, anti-p21, or anti-GAPDH antibodies. (**e** and **f**) HLF and Hep3B were treated with Mg132 (20 μg/ml) for 4 h, then lysed with IP lysis/wash buffer with protease inhibitor, phosphatase inhibitor and 10 μM N-ethylmaleimide. p21 was immunoprecipitated with an anti-p21 antibody, and the immune-precipitates were probed with anti-ubiquitin, anti-FBXO22, anti-p21 and anti-GAPDH antibodies. (**g**) HEK293T cells transfected with Flag-p21, HA-ubiquitin, Myc-FBX022 and Myc-FBX022^ΔF-BOX^ in combination were treated with Mg132 (20 μg/ml) for 4 h, then lysed with IP lysis/wash buffer with protease inhibitor, phosphatase inhibitor and 10 μM N-ethylmaleimide. Flag-p21 was immunoprecipitated with a Flag antibody, and the immune-precipitates were probed with anti-HA, anti-Flag, anti-Myc and anti-GAPDH antibodies

A major function of F-box proteins is the ubiquitination of their target substrates. Since the results confirmed that FBXO22 regulates the protein levels of p21 and interacts with it, we next investigated whether FBXO22 could ubiquitinate p21 protein in vitro. Cells were treated with cycloheximide (CHX) to inhibit protein biosynthesis, and protein extracts obtained at the indicated time points were analyzed. We found that knockdown of FBXO22 in HLF cells resulted in a significantly extended half-life of p21 (Fig. 5a). Conversely, the overexpression of FBXO22 in Hep3B and LM3 cells profoundly decreased the half-life of the p21 protein (Fig. 5b and Additional file 2: Figure S2A). These results indicated that FBXO22 mediates the degradation of p21 protein in HCC cells. Furthermore, the effect of FBXO22 on p21 could be blocked by the proteasome inhibitor MG132 (Figs. 5c and d; Additional file 2: Figures S2B and S2C). This further suggested that the regulation may be related to the ubiquitination process. Therefore, we implemented a ubiquitination experiment and found that FBXO22 downregulation was accompanied by reduced ubiquitination in HLF and HepG2 cells (Fig. 5e and Additional file 2: Figures S2D, S3A and S3B), while the overexpression of FBXO22 was accompanied by enhanced ubiquitination in LM3 and Hep3B cells (Fig. 5f and Additional file 2: Figures S2E, S3C, and S3D). Therefore, p21 can be degraded by FBXO22 via a ubiquitination-mediated proteasome-dependent mechanism.

FBXO22 contains an N-terminal F-box domain and a C-terminal FIST_C (F-box and intracellular signal transduction, C-terminal) domain (Additional file 2: Figure S2F). To identify which domain of FBXO22 mediates the ubiquitination p21, we generated Myc-FBXO22 truncated constructs and an N-terminal deletion (amino acids 100–403) mutant (Δ^F-BOX^). To do this, HEK293T cells were transfected with Flag-p21, HA-ubiquitin, Myc-FBXO22 and Myc-FBXO22^ΔF-BOX^. The sequential IP and immunoblotting analysis showed a significant increase of polyubiquitinated p21 protein in the cells transfected with wild-type FBXO22, as well as a decrease in the cells transfected with the N-terminal deletion mutant (Δ^F-BOX^) (Fig. 5g and Additional file 3: Figure S3E, respectively). These results suggest that the ubiquitination of p21 by FBXO22 is mediated by its F-box domain (amino acids 1–100).

### FBXO22 promotes HCC cell growth by downregulating the levels of p21 and affects cell cycle and apoptosis induced by DNA damage in vitro

**Fig. 6:**
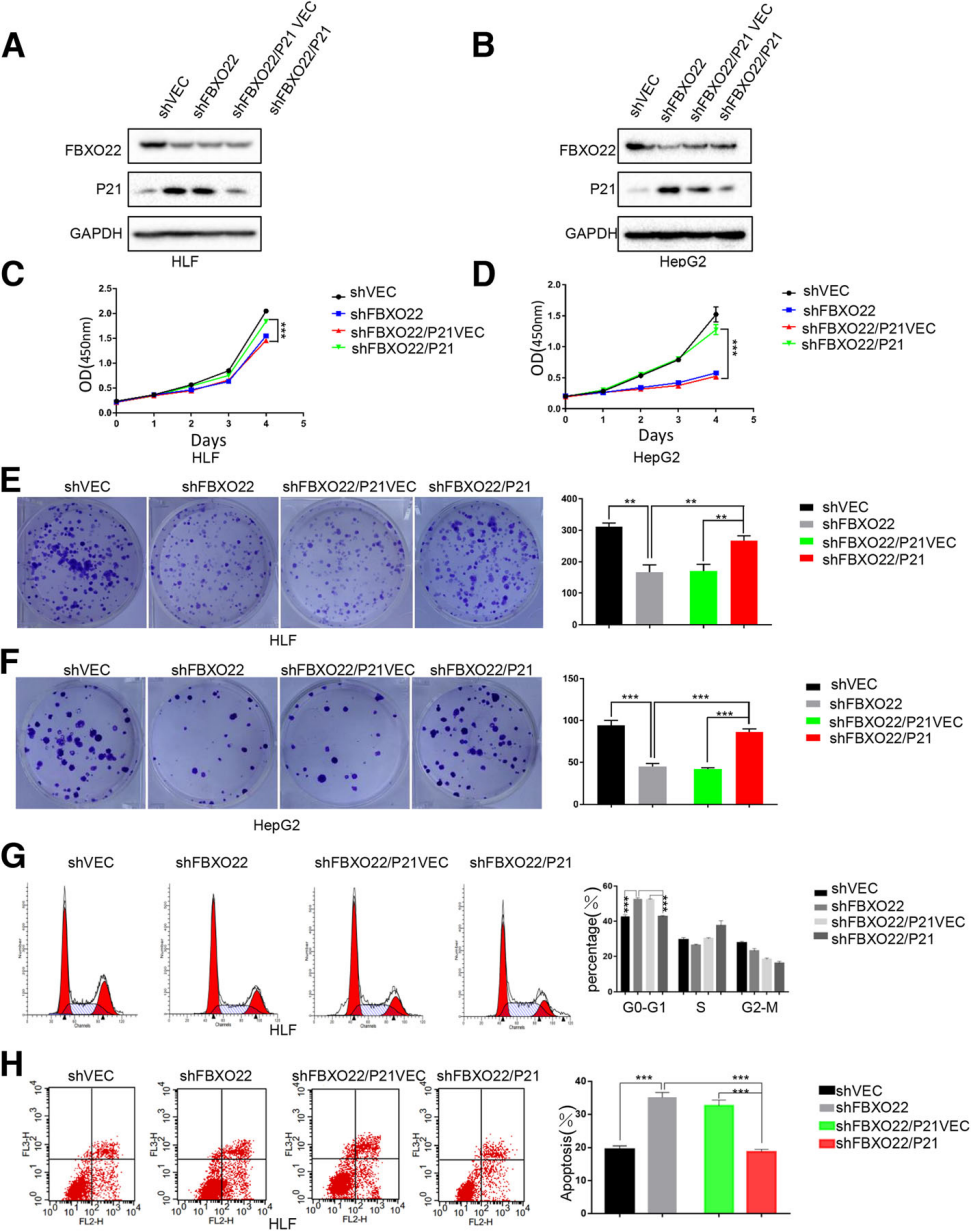
FBXO22 promotes HCC cell growth by down-regulating the levels of p21 and affects cell cycle and apoptosis induced by DNA damage in vitro. (**a** and **b**) FBXO22-depleted HLF and HepG2 cells were transfected with the indicated constructs, total protein was extracted and subjected to western blotting using anti-FBXO22, anti-p21, or anti-GAPDH antibodies. Knockdown of p21 expression effectively promoted cell growth (**c** and **d**) and foci formation (**e** and **f**). The results are expressed as the means ± standard error of the mean (SEM) of three independent experiments. (**e**) HLF cells infected with the indicated lentiviral shRNAs were transfected with the indicated constructs for 24 h. Cells were stained with propidium iodide and analyzed using flow cytometry. The error bars represent the mean ± SD of three independent experiments. (**f**) HLF cells were infected with the indicated lentiviral shRNAs. Cells were then treated with either 0.2 μM doxorubicin (Dox) for 48 h, followed by flow cytometry analysis. The error bars indicate the mean ± SD of three in- dependent experiments

To further determine whether FBXO22 performs its function through the ubiquitination of p21, we ectopically knocked down p21 in FBXO22-delepted HLF and HepG2 cells and verified the protein depletion by western blot analysis (Fig. 6a and b, respectively). Importantly, the ectopic knockdown of p21, but not the empty vector, significantly restored the viability (*p*<0.01, one-way ANOVA) of HLF and HepG2 cells with low expression of FBXO22 (Fig. 6c and d, respectively). In addition, colony formation assays also showed that the foci formation frequency was obviously restored when p21 was knocked down in FBXO22-delepted HLF (*p*<0.05, one-way ANOVA) and HepG2 cells (*p*<0.01, one-way ANOVA), compared with the control cells (Fig. 6e and f, respectively). Collectively, these data suggest that FBXO22 promotes HCC cell growth by downregulating the levels of p21.

Since p21 regulates cell cycle progression in G1 phase, we speculate that FBXO22 affects cell cycle progression from G1 to S phase through p21. We performed cell cycle detection on HLF cell lines that knocked down shFBXO22 with p21 rescue (Fig. 6g), and found that when FBXO22 was knocked down, G1 to S phase were blocked, and when p21 was rescued, the percentage of S phase cells increased (*p*<0.01, one-way ANOVA), indicating FBXO22 mediating the transition from G1 to S depends on p21.

To investigate the effect of FBXO22 on apoptosis induced by a DNA damaging agent, HLF cell lines that knocked down FBXO22 with p21 rescue were treated with doxorubicin (Fig. 6h). The percentage of apoptotic cells was measured using flow cytometry with PI (propidium iodide) staining. Compared with the control cells, FBXO22-depleted HLF cells exhibited a significant increase in the levels of apoptosis after a 24 h treatment with doxorubicin. Interestingly, when p21 was rescued, the percentage of apoptosis cells decreased (*p*<0.01, one-way ANOVA). Collectively, these data showed that FBXO22 knockdown sensitized cells to DNA damage-induced apoptosis by promoting p21 accumulation.

### Correlation between FBXO22 and p21 in clinical samples

**Fig. 7:**
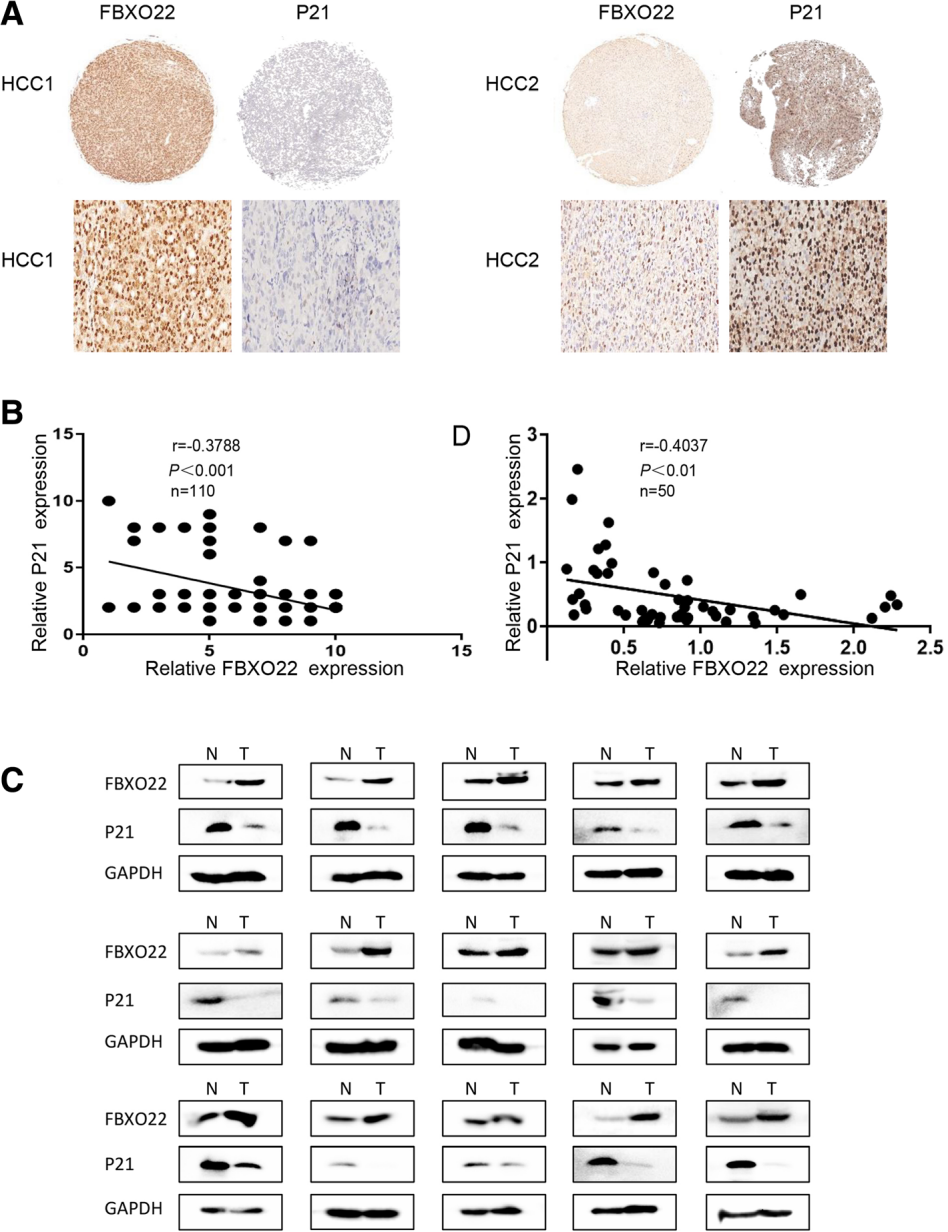
Correlation between FBXO22 and p21 in clinical samples (**a**) Representative images of IHC staining with anti-FBXO22 and anti-p21 (400×, magnification). (**b**) The expression of FBXO22 was inversely correlated with that of p21. (**c**) Western blot analysis of FBXO22 expression in HCC and non-cancerous tissues. GAPDH was used as a loading control. (**d**) The expression of FBXO22 was negatively correlated with p21

To investigate the correlation between FBXO22 and p21 in HCC, we analyzed the expression of FBXO22 and p21 by IHC on a tissue microarray containing 110 pairs of liver cancer samples with clinical follow-up information. The results showed that expression of FBXO22 was negatively correlated with p21 in the clinical samples (r = − 0.3788, *P*<0.001, Pearson correlation, Figs.7A and 7b, respectively). In addition, we also determined protein level of FBXO22 and p21 expression in 50 pairs of primary HCC and an adjacent non-tumoral liver tissue. Western blot analysis showed that the expression level of FBXO22 in the tumor group was significantly higher than that in the non-tumor liver group (30 of 50 cases) (Fig. 7c and Additional file 4: Figure S4, respectively). Correlation analysis also showed that FBXO22 was negatively correlated with p21 in the clinical samples (r = − 0.4037, *P*<0.01, Pearson correlation, Fig. 7d). These results indicated that the expression of FBXO22 and p21 was negatively correlated in clinical samples.

## Discussion

In this study, we explored the oncogenicity of FBXO22 in the pathogenesis and progression of HCC. Overexpression of FBXO22 was detected in 61.8% (68/110) of HCC patients and was significantly correlated with serum AFP (*p* = 0.003), tumor size (*p* = 0.019), and vascular invasion (*p* = 0.031). On univariate survival analysis, serum AFP, tumor size, BCLC stage, TNM stage, differentiation, vascular invasion, and expression of FBXO22 were associated with OS. Multivariate analysis showed that tumor size and the expression of FBXO22 were independent prognostic indicator of OS. The overall survival and disease-free survival of patients with high expression of FBXO22 were significantly shorter than those of patients with low expression of FBXO22. Taken together, these data strongly suggest that FBXO22 functions as an oncogene and plays an important role in the development of HCC. Especially, FBXO22 can be acted as an independent prognostic indicator of OS.

We investigated the oncogenic mechanism of FBXO22 by functional studies in vivo and in vitro. Knockdown of FBXO22 inhibited cell growth in vitro and tumor formation in nude mice, while overexpression of FBXO22 increased cell viability. Because FBXO22 plays an important role during tumor progression, we tested the levels of proteins related to signaling pathways and the cell cycle. Knockdown or overexpression of FBXO22 obviously influenced the endogenous p21 protein levels. However, up-regulation of FBXO22 only had a slight impact on average p21 mRNA levels. These results suggest that the regulation of p21 by FBXO22 is mediated by post-transcriptional modification in HCC. A previous study showed that FBOXO22 interacts with p53, which can regulate the expression of p21 [10]. We therefore determined whether FBXO22 interacted with p21. Overexpression of FBXO22 was accompanied by enhanced ubiquitination, while its downregulation was accompanied by reduced ubiquitination, which was mediated by the F-box domain.

The cyclin-dependent kinase inhibitor p21 (also named p21WAF1/Cip1 or CDKN1a) is a cell-cycle inhibitor controlled by p53 dependently or independently [19]. Previous studies have indicated that p21 holds a central position in the response to DNA damage and cell cycle regulation primarily via G1 or G2 phase arrest in response to a variety of stress stimuli [20]. Our data showed FBXO22 mediated the transition from G1 to S phase depending on p21 and that FBXO22 knockdown sensitized cells to DNA damage-induced apoptosis by promoting p21 accumulation. A recent study demonstrated that FBXO22 ubiquitinates and destabilizes p53 [10], which was consistent with our findings. Further, we found that FBXO22 interacts with p21 as well as p53. Overexpression or knockdown of FBXO22 affected p21 protein levels via the ubiquitination pathway, suggesting a new mechanism linking FBXO22 dysregulation with HCC. However, it is still unclear whether the regulation of p21 by FBXO22 depends on p53. Kim. J et al. reported that p53 and p21 mutually interact [17]. Moreover, Bcl-2 interacts with p53 or p21 separately, while invasion or cell death is regulated by p53/p21 complexes through the degradation of Bcl-2 protein [18]. Similarly, we confirmed that FBXO22 interacts with p53 or p21 individually, which supports the hypothesis that FBXO22 might regulate cell proliferation by targeting the degradation of the p53/p21 complex in HCC.

Ubiquitination reactions play important roles in development, but when this process goes wrong, unregulated cell growth occurs, leading to the development of tumors [21, 22]. The short-lived protein p21 is regulated mainly by posttranslational modifications such as phosphorylation and ubiquitin-dependent proteolysis. In terms of the ubiquitination-dependent pathway, it has been identified that p21 can be ubiquitylated and degraded by the three E3 ubiquitin ligase complexes SCF^skp2^, CRL4^CDT2^ and APC/C^CDC20^ at specific stages of the cell cycle [23–26]. Furthermore, SCF^Fbxo22^ ubiquitylated p53 and formed a complex with KDM4A, which is an E3 ubiquitin ligase that targets methylated p53 and regulates key senescence processes [10]. SCF^skp2^ functions in p21 degradation during both the G1/S transition and the S phase [21], whereas CRL4^CDT2^ promotes the degradation of p21 specifically during the S-phase of the cell cycle [24]. During mitosis, p21 degradation is also driven by the APC/C^CDC20^ complex [27]. Therefore, we speculated that FBXO22 targets p21 for ubiquitin-mediated degradation via the E3 ubiquitin ligase complex. However, further studies are needed to fully understand the detailed mechanism.

In summary, our study reveals a novel mechanism by which FBXO22 acts as an oncogene in the pathogenesis and progression of HCC by mediating the ubiquitination and degradation of p21. Our findings present a new perspective for understanding the development of HCC and can provide new targets for the treatment and management of this deadly cancer.

## Conclusion

We demonstrated that FBXO22 regulates cell proliferation by degrading p21 through ubiquitination, which promoted tumorigenesis in HCC by experiments in vitro and in vivo.

## Additional files

Additional file 1: **Figure S1.** FBXO22 promotes proliferation and tumorigenesis of HCC cells in vitro and effect of FBXO22 on the cell cycle and signaling pathways (a and b) FBXO22 promote the proliferation of HCC cells in vitro. Transfection with scrambled shRNA (shvec) was used as negative control. Knockdown of FBXO22 expression effectively inhibited cell growth (a) and foci formation (b). (c) The tumor volume at different days of the xenograft tumors. (d) Effect of FBXO22 on cell cycle and signaling pathways. HLF and HepG2 cells were transfected with the indicated constructs, total protein was extracted and subjected to western blotting using the indicated antibodies. (e) Effect of FIST-N and FIST-C domains on p21. 293 T cells were transfected with the indicated constructs, total protein was extracted and subjected to western blotting using the indicated antibodies. (JPG 754 kb)

Additional file 2: **Figure S2.** FBX022 ubiquitinates p21 and F-box domain mediates the process (a) LM3 cells were treated with CHX (10 μM), collected at the indicated time points, and immunoblotted for FBXO22, p21 and GAPDH. Quantification of the p21 levels relative to GAPDH expression is shown. (b and c) HepG2 and LM3 cells were treated with Mg132 (10 μg/ml) for 4 h, total protein was extracted and subjected to western blotting using anti-FBXO22, anti-p21, or anti-GAPDH antibodies. (d and e) HepG2 and LM3 were treated with Mg132 (20 μg/ml) for 4 h, then lysed with IP lysis/wash buffer with protease inhibitor, phosphatase inhibitor and 10 μM N-ethylmaleimide. p21 was immunoprecipitated with an anti-p21 antibody, and the immune-precipitates were probed with anti-FBXO22, anti-ubiquitin and anti-p21 antibodies. (f) schematic representation of the domain structure of FBXO22 (JPG 608 kb)

Additional file 3: **Figure S3.** FBX022 ubiquitinates p21 via the F-box domain HLF (a), HepG2 (b), Hep3B (c) and LM3 cells (d) were treated with Mg132 (20 μg/ml) for 4 h, then lysed with IP lysis buffer with protease inhibitor, phosphatase inhibitor and 10 μM N-ethylmaleimide. Total protein was extracted and subjected to western blotting using anti-FBXO22, anti-p21, anti-ubiquitin or anti-GAPDH antibodies. (e) HEK293T cells transfected with Flag-p21, HA-ubiquitin, Myc-FBX022 and Myc-FBX022^ΔF-BOX^ in combination were treated with Mg132 (20 μg/ml) for 4 h, then lysed with IP lysis buffer with protease inhibitor, phosphatase inhibitor and 10 μM N-ethylmaleimide. Total protein was extracted and subjected to western blotting using anti-HA, anti-Myc, anti- Flag or anti-GAPDH antibodies. (JPG 572 kb)

Additional file 4: **Figure S4.** Correlation between FBXO22 and p21 in clinical samples western blot analysis of FBXO22 and p21expression in HCC and non-cancerous tissues. GAPDH was used as a loading control. (JPG 649 kb)

## Abbreviations

CCK8: Cell Counting Kit-8
CHX: Cycloheximide
DFS: Disease-free survival
H&E: Hematoxylin and eosin
HCC: Hepatocellular carcinoma
IHC: Immunohistochemistry
OD: Optical density
OS: Overall survival
PCR: Polymerase Chain Reaction
