# Supplementary figures and images for: FBXO22 promotes the development of hepatocellular carcinoma by regulating the ubiquitination and degradation of p21
Source: J Exp Clin Cancer Res. 2019 Feb 26;38:101. doi: 10.1186/s13046-019-1058-6 (PMC6390379; doi:10.1186/s13046-019-1058-6)

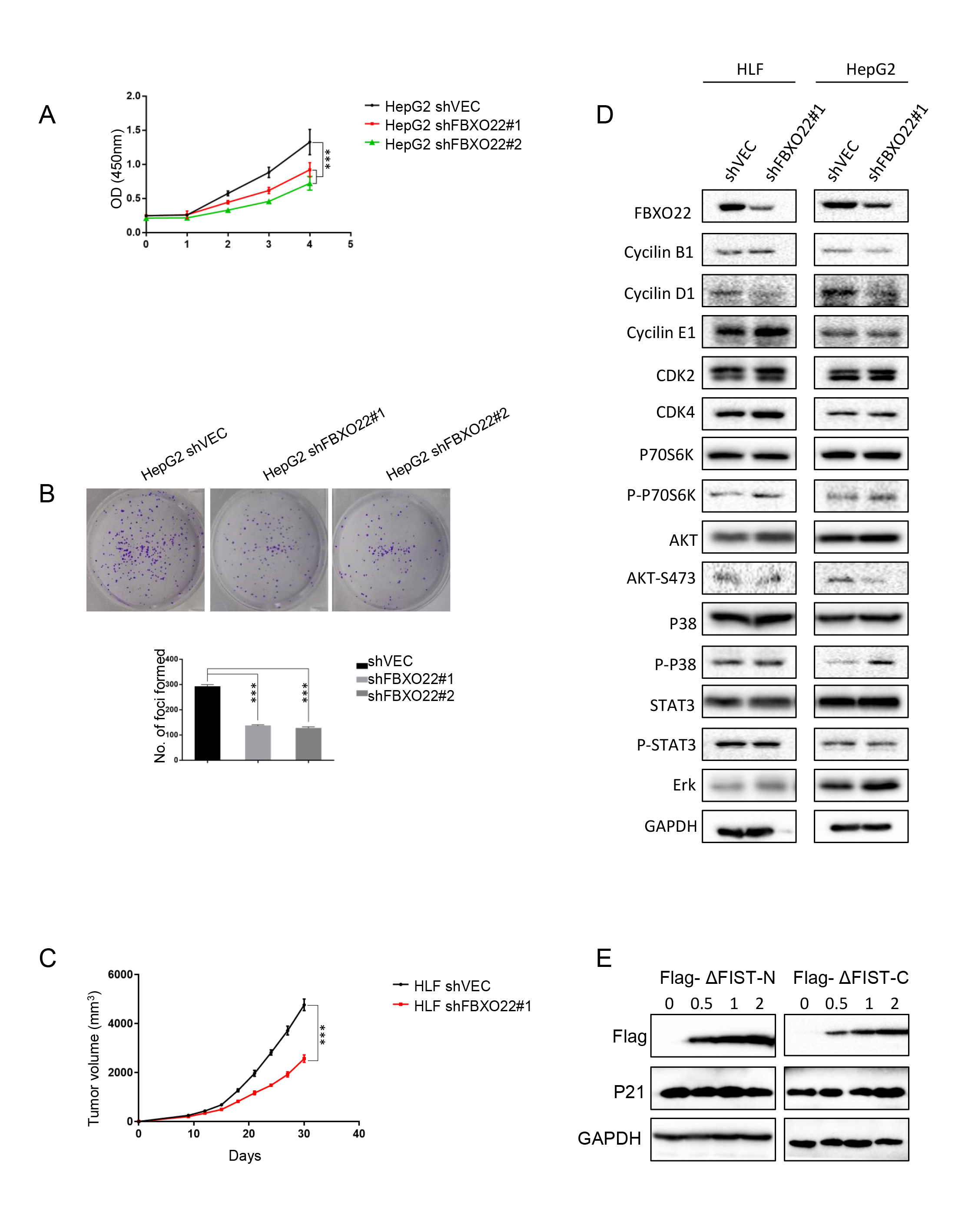

Supplement: Supplementary file 1 — Figure S1. FBXO22 promotes proliferation and tumorigenesis of HCC cells in vitro and effect of FBXO22 on the cell cycle and signaling pathways (a and b) FBXO22 promote the proliferation of HCC cells in vitro. Transfection with scrambled shRNA (shvec) was used as negative control. Knockdown of FBXO22 expression effectively inhibited cell growth (a) and foci formation (b). (c) The tumor volume at different days of the xenograft tumors. (d) Effect of FBXO22 on cell cycle and signaling pathways. HLF and HepG2 cells were transfected with the indicated constructs, total protein was extracted and subjected to western blotting using the indicated antibodies. (e) Effect of FIST-N and FIST-C domains on p21. 293 T cells were transfected with the indicated constructs, total protein was extracted and subjected to western blotting using the indicated antibodies. (JPG 754 kb) [file 13046_2019_1058_MOESM1_ESM.jpg]

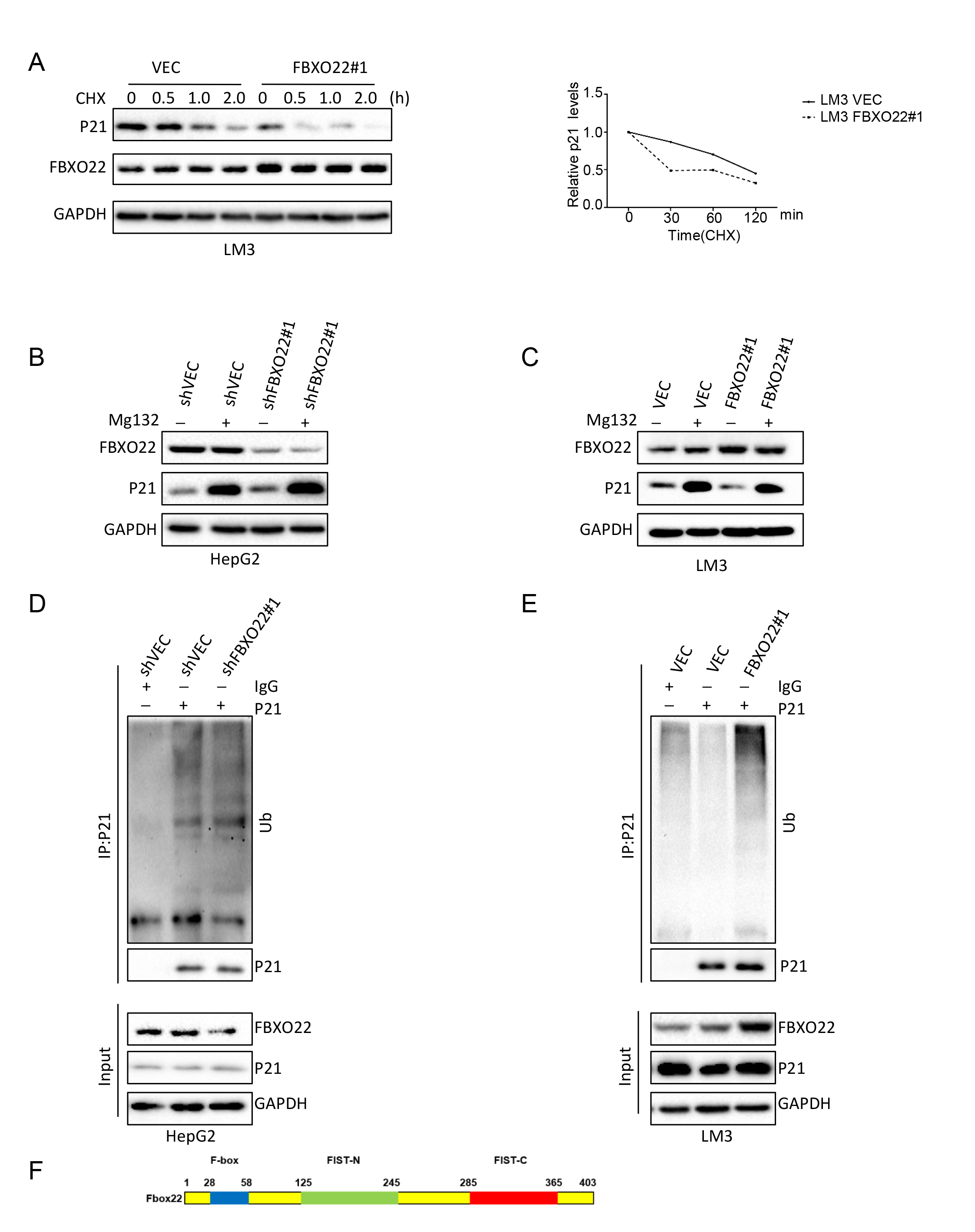

Supplement: Supplementary file 2 — Figure S2. FBX022 ubiquitinates p21 and F-box domain mediates the process (a) LM3 cells were treated with CHX (10 μM), collected at the indicated time points, and immunoblotted for FBXO22, p21 and GAPDH. Quantification of the p21 levels relative to GAPDH expression is shown. (b and c) HepG2 and LM3 cells were treated with Mg132 (10 μg/ml) for 4 h, total protein was extracted and subjected to western blotting using anti-FBXO22, anti-p21, or anti-GAPDH antibodies. (d and e) HepG2 and LM3 were treated with Mg132 (20 μg/ml) for 4 h, then lysed with IP lysis/wash buffer with protease inhibitor, phosphatase inhibitor and 10 μM N-ethylmaleimide. p21 was immunoprecipitated with an anti-p21 antibody, and the immune-precipitates were probed with anti-FBXO22, anti-ubiquitin and anti-p21 antibodies. (f) schematic representation of the domain structure of FBXO22 (JPG 608 kb) [file 13046_2019_1058_MOESM2_ESM.jpg]

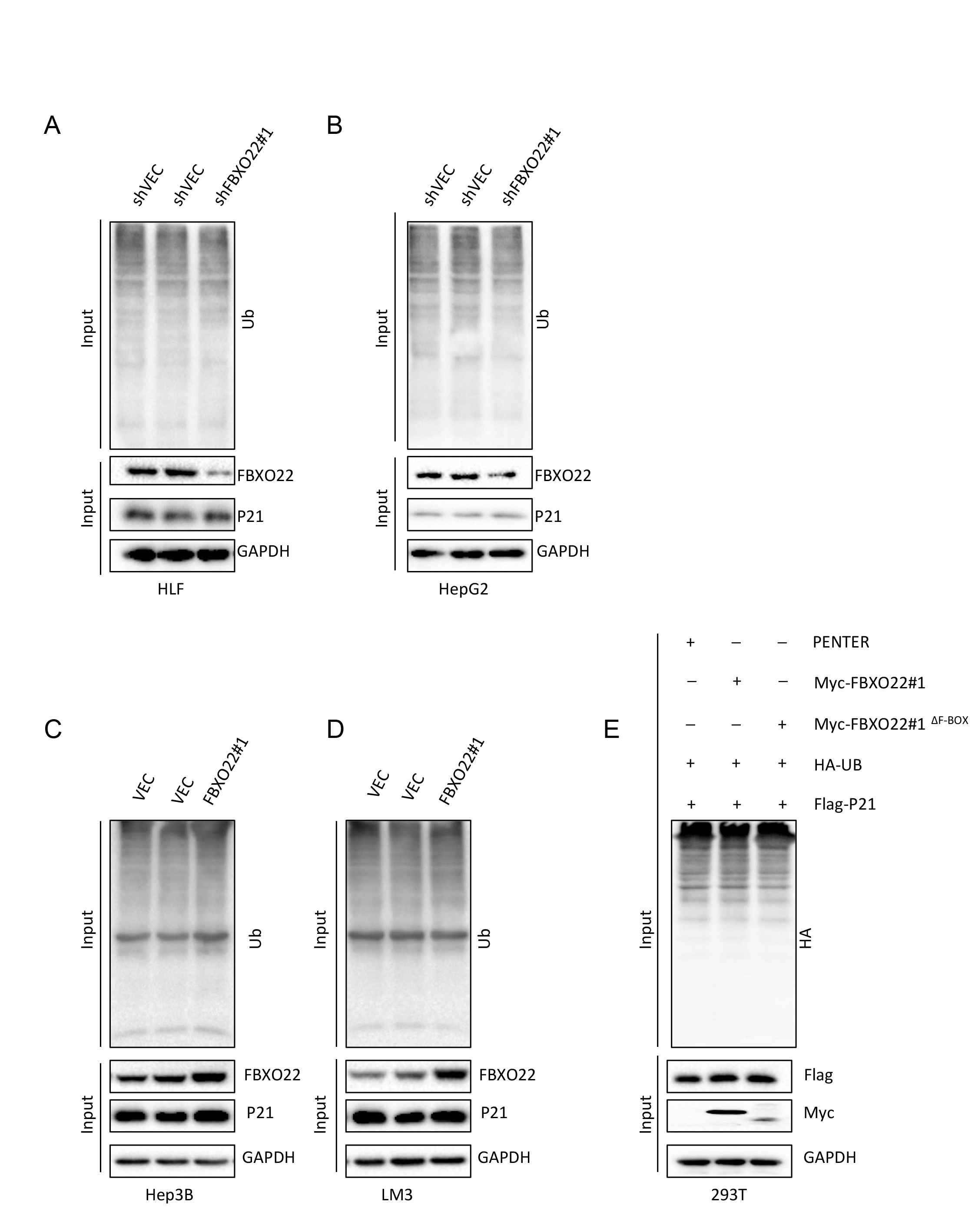

Supplement: Supplementary file 3 — Figure S3. FBX022 ubiquitinates p21 via the F-box domain HLF (a), HepG2 (b), Hep3B (c) and LM3 cells (d) were treated with Mg132 (20 μg/ml) for 4 h, then lysed with IP lysis buffer with protease inhibitor, phosphatase inhibitor and 10 μM N-ethylmaleimide. Total protein was extracted and subjected to western blotting using anti-FBXO22, anti-p21, anti-ubiquitin or anti-GAPDH antibodies. (e) HEK293T cells transfected with Flag-p21, HA-ubiquitin, Myc-FBX022 and Myc-FBX022ΔF-BOX in combination were treated with Mg132 (20 μg/ml) for 4 h, then lysed with IP lysis buffer with protease inhibitor, phosphatase inhibitor and 10 μM N-ethylmaleimide. Total protein was extracted and subjected to western blotting using anti-HA, anti-Myc, anti- Flag or anti-GAPDH antibodies. (JPG 572 kb) [file 13046_2019_1058_MOESM3_ESM.jpg]

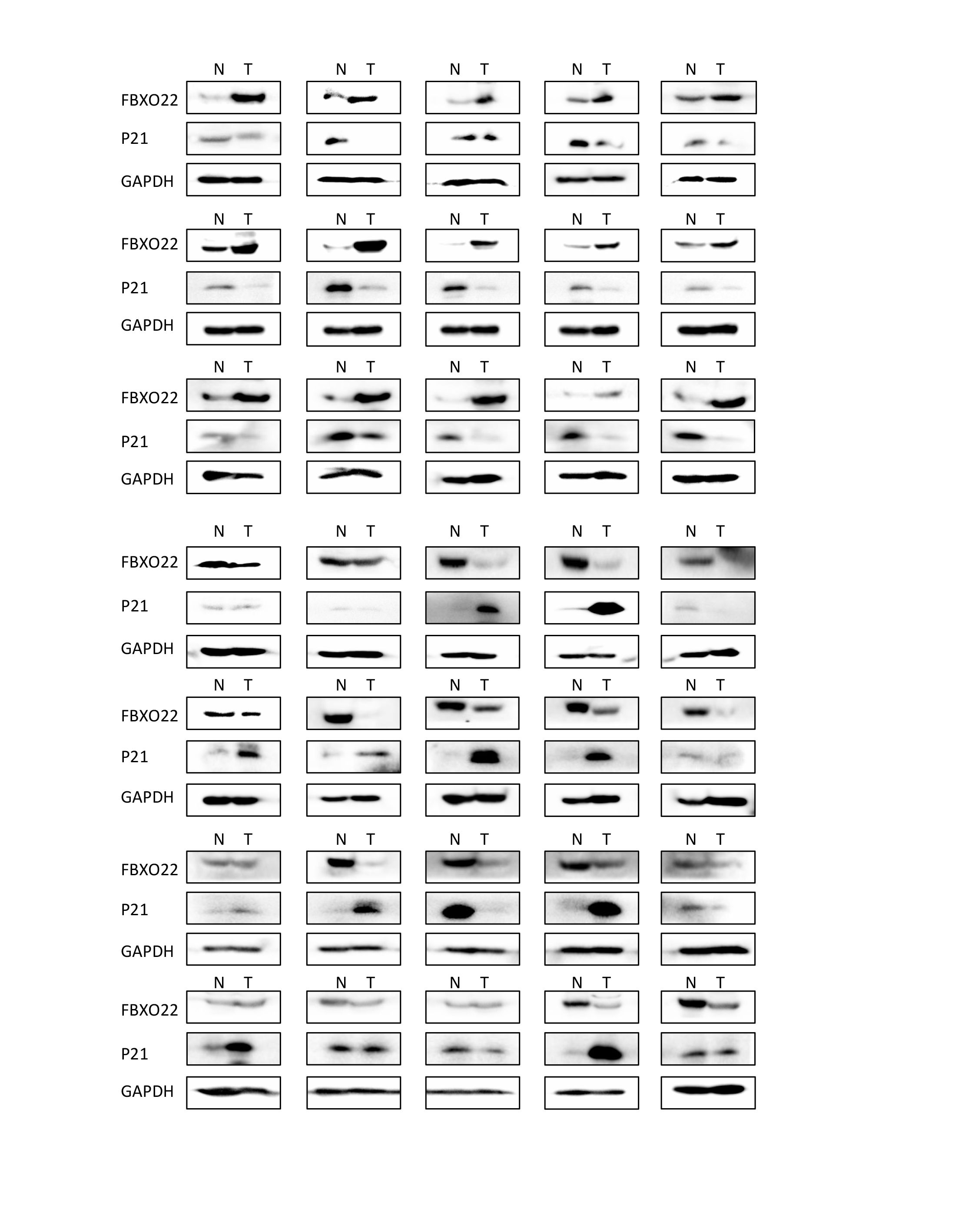

Supplement: Supplementary file 4 — Figure S4. Correlation between FBXO22 and p21 in clinical samples western blot analysis of FBXO22 and p21expression in HCC and non-cancerous tissues. GAPDH was used as a loading control. (JPG 649 kb) [file 13046_2019_1058_MOESM4_ESM.jpg]
